# Broadband bifunctional Luneburg–Fisheye lens based on anisotropic metasurface

**DOI:** 10.1038/s41598-020-77270-0

**Published:** 2020-11-23

**Authors:** Jiaqing Chen, Yongjiu Zhao, Lei Xing, Zheng He, Luyang Sun

**Affiliations:** College of Electronic and Information Engineering, Nanjing University of Aeronautics and Astronautics, Nanjing, 211100 China

**Keywords:** Materials science, Optics and photonics, Physics

## Abstract

Luneburg lenses and Maxwell fisheye lenses possess distinct properties of focusing, well beyond conventional lenses made of uniform materials. In this paper, a planar broadband bifunctional Luneburg-fisheye lens synthesized by gradient anisotropic metasurface is proposed. The proposed anisotropic metasurface is formed by non-resonant anisotropic cells, so that it can independently realize the equivalent gradient refractive indexes of Luneburg lens and Maxwell fisheye lens along orthogonal directions in a broad band, respectively. To verify the performance of the bifunctional lens, a prototype associated with a feeding log-periodic dipole antenna has been fabricated. Experimental results show that the proposed lens functions well over a wide frequency range with high efficiency and low profile, which coincides well with theoretical predictions and simulated results. It is expected that the proposed design will facilitate the applications of multifunctional metadevices in microwave and optical ranges.

## Introduction

In the past decade, metasurfaces, by virtue of their fantastic physical characteristics associated with ultrathin configurations, have attracted an extensive interest in the fields of microwave, optics, and even acoustics^1–3^. Following the proposal of the generalized Snell’s laws of reflection and refraction^4,5^, gradient metasurfaces have proven to be an unprecedented approach to manipulating the propagation of electromagnetic (EM) waves. Since then, a range of revolutionary functionalities have been realized, such as beam steering^6–8^, invisibility cloaks^9^, polarization rotators^10^, vortex phase plates^11,12^, and flattened gradient lenses^13–15^.

As alternatives to conventional lenses, gradient refractive index (GRIN) lenses constructed by inhomogeneous artificially structured metasurfaces can achieve significant reductions in volume and weight, facilitating the integration with other microwave or optical devices^15^. Furthermore, benefiting from the elimination of wave font aberration, metasurface-based gradient lenses are more capable for highly-directional emission with low loss^13,16–20^. Among the GRIN lenses, Luneburg lens and Maxwell Fisheye lens have come into prominence recently and become more and more indispensable in collimating lights, wide-angle cameras, and communication systems due to their distinct properties of focusing^21^.

Luneburg lens is a typical GRIN lens with spherical symmetry, whose refractive index varies gradually from at the center to 1 on the outermost shell. Theoretically, it is able to guide the incoming plane wave from arbitrary directions to a focal point on the opposite surface of the lens without aberration (or vice versa). Similarly, Maxwell Fisheye lens is a spherically symmetric GRIN lens as well, whose refractive index decreases radially from 2 at the center to 1 on the perimeter in general. It can focus the radiation emitted from a point source to a diametrically opposite point on the lens rim.

Despite the ease of fabrication and superior performance, most of the Luneburg lenses or Maxwell fisheye lenses designed previously by metamaterials still suffer from bulk configurations^22–26^. Planar Luneburg lenses or fisheye lenses can effectively lower the profile of the whole structure, while maintaining the radiation characteristics. However, most of the flat metalenses in contemporary works could hardly adjust the distributions of the refractive index along two polarization directions independently^27–32^, namely that most of them are isotropic and serving as single-function devices. Resulting from the demand for miniaturization and multifunction of microwave or optical devices, integration of multiple functions has been brought into focus. As we know, anisotropic metasurfaces (AMs) can manipulate orthogonally polarized waves independently with high efficiency^33^, which would be a promising candidate for the design of multifunctional metalens. Hence, gradient anisotropic metasurface (GAM), which can achieve different gradient distributions of refractive index along orthogonal directions, is evolved. By adjusting the geometrical parameters of the GAMs, bifunctional metalenses, combining the function of a Luneburg lens and that of a fisheye lens, are presented^21,34,35^. While, the bandwidths of the lenses mentioned above are extremely restricted because of the intrinsic resonance characteristics of the AMs and the coupling between adjacent cells.

In this paper, a broadband bifunctional Luneburg-fisheye lens constructed by gradient anisotropic metasurface (GAM) is proposed, which characters as a Luneburg lens viewing from the horizontal axis in the frequency range of 2 to 8 GHz (relative bandwidth of 120%), while as a Maxwell fisheye lens viewing from the vertical axis in the band of 5 to 7 GHz (relative bandwidth of 33.3%). The proposed design of the Luneburg-fisheye lens stands out from the aforementioned designs in three aspects. First, the proposed metalens can achieve a bifunctional response of a Luneburg lens and a fisheye lens over a broad band far beyond the existing designs. The constitutive GAM is synthesized by non-resonant anisotropic cells applying with quasi-conformal mapping method, so that it can independently regulate the inhomogeneous refractive-index distributions in two orthogonal directions. Second, the bifunctional lens is designed to be flattened with compact size and low loss, resulting in easy integration with other devices. Third, what needs to be highlighted is that this anisotropy-based approach is not limited to a combination of the two lenses. It can be flexibly extended to the realization of other broadband bifunctional metadevices, such as Luneburg-Eaton lens, Eaton-fisheye lens, and so on.

## Results

### Design and analysis of the anisotropic unit cell

**Figure 1:**
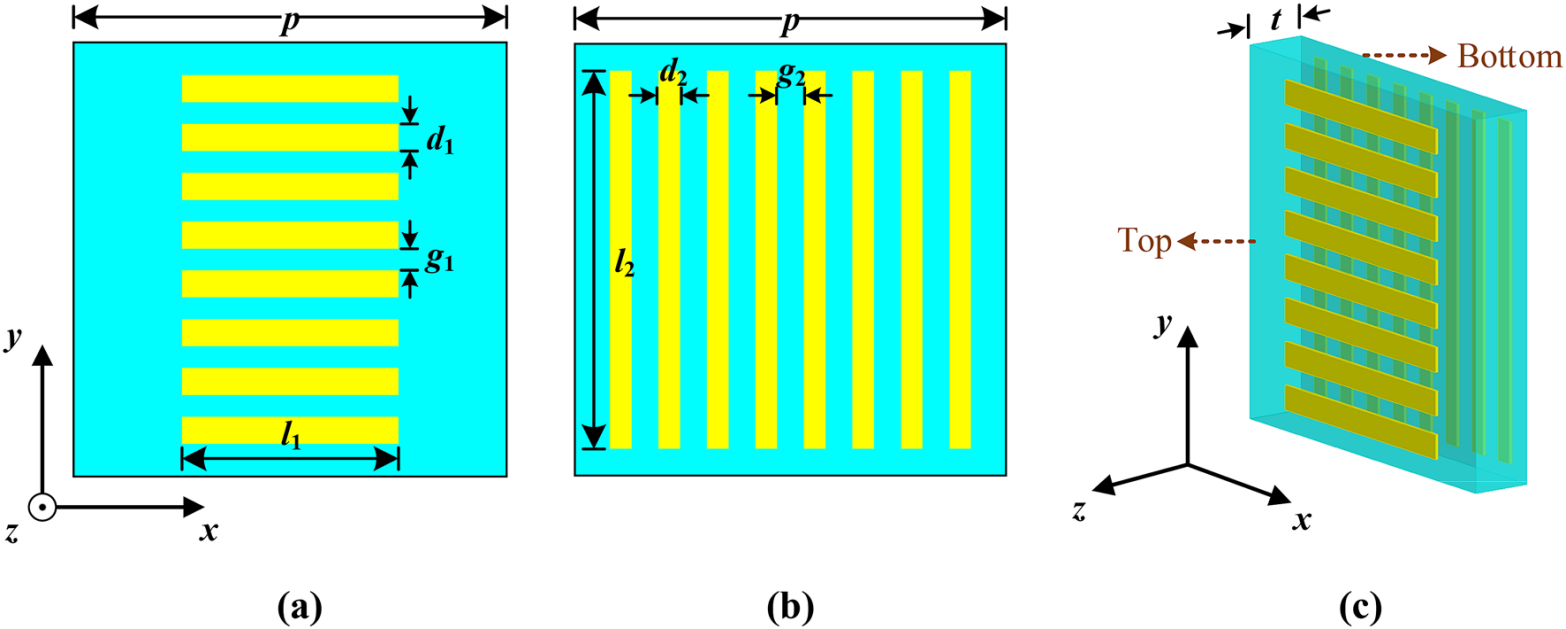
Configuration of the proposed anisotropic cell. (**a**) Top view. (**b**) Bottom view. (**c**) Perspective view.

The configuration of the proposed anisotropic unit cell used for the synthesis of the bifunctional lens is demonstrated in Fig. 1. The anisotropic cell consists of two mutually orthogonal parallel-line arrays. The two sets of the metallic array are attached separately on both sides of a commercial F4B substrate (*ε*_*r*_ = 2.2, tan *δ* = 0.001) with a thickness of *t* = 1 mm (0.027*λ* at 8 GHz). Considering the operating band of the metalens, the periodicity *p* of the anisotropic cell is set as 6 mm.

**Figure 2:**
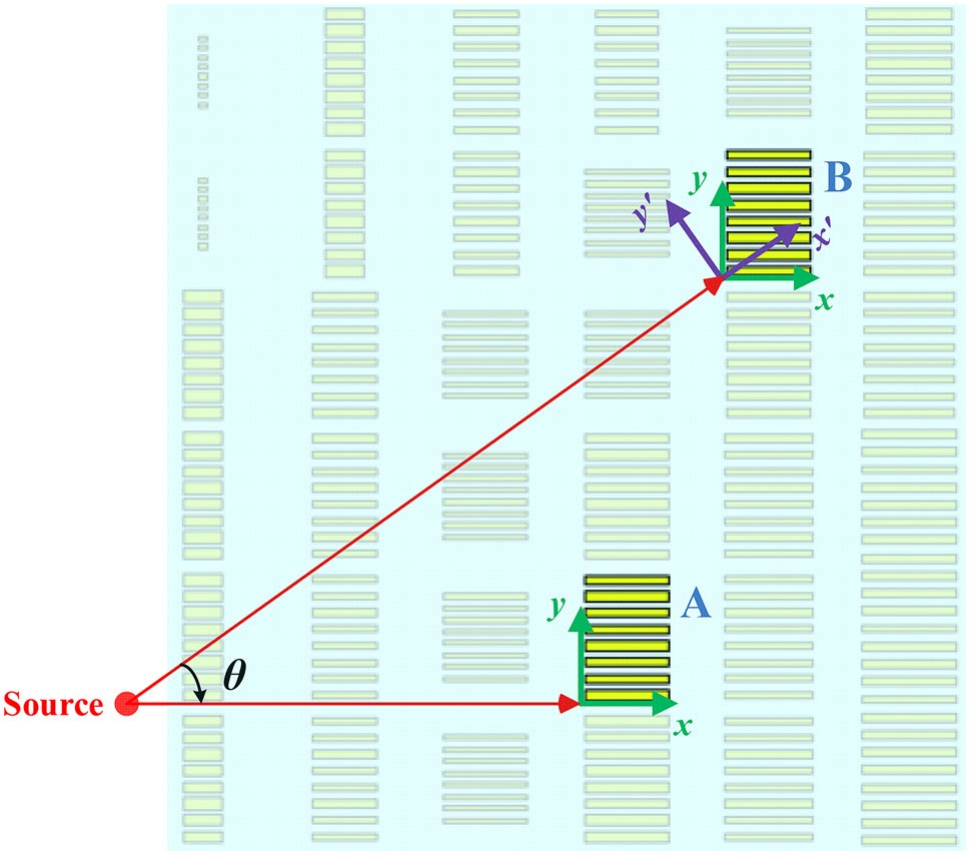
A fraction of the synthesized lens based on anisotropic cells (Top View).

As mentioned above, it is extremely difficult to realize a bifunctional lens with isotropic materials. Here, we will discuss the feasibility of realizing a Luneburg-fisheye lens with anisotropic metasurface (AM). Suppose that a point source is located on *xoy*-plane to illuminate the synthesized metalens, which is inhomogeneous, as shown in Fig. 2. When the EM wave emitted from the point source travels along *x* axis, the refractive index of the cell block is denoted as *n*_*x*_ (like Unit A). Similarly, when the EM wave propagates along *y* axis, the refractive index of the cell block is denoted as *n*_*y*_. In the case of oblique incidence with an angle of *θ* relative to the *x*-axis, we rotate the former coordinates counterclockwise by the angle of *θ* so that a new coordinate system can be formed, marked by *x*’-*y*’ axes, as depicted in Unit B. The coordinate transformation resulting from the rotation can be expressed as follows:

1 $$\left\{ {\begin{array}{*{20}c} {x^{\prime} = x\cos \theta + y\sin \theta } \\ {y^{\prime} = y\cos \theta - x\sin \theta } \\ \end{array} } \right.$$

With respect to the new coordinate system, the refractive index tensor of the cell block can be calculated by the following expression.

2 $$\left\{ \begin{gathered} n_{x^{\prime}x^{\prime}} = n_{x} \cos^{2} \theta + n_{y} \sin^{2} \theta \hfill \\ n_{y^{\prime}y^{\prime}} = n_{x} \sin^{2} \theta + n_{y} \cos^{2} \theta \hfill \\ n_{x^{\prime}y^{\prime}} = n_{y^{\prime}x^{\prime}} = (n_{y} - n_{x} )\sin \theta \cos \theta \hfill \\ \end{gathered} \right.$$

As we know, to realize a bifunctional metalens, the metasurface should be anisotropic in orthogonal directions (i.e. *x*- and *y*-axis). While, the AM is also expected to remain nearly isotropic along each axis under the circumstance of oblique incidence. As indicated by expression (2), when the incident angle *θ* is small, *n*_*x*’*x*’_ and *n*_*y*’*y*’_ is approximately equal to *n*_*x*_ and *n*_*y*_, respectively. Furthermore, the cross-polarization components *n*_*x*’*y*’_ and *n*_*y*’*x*’_ are approaching zero in this case. However, if *θ* is getting larger, the preceding conclusions will only be applicable when the values of *n*_*x*_ and *n*_*y*_ are close to each other. According to the previous descriptions of the Luneburg lens and Maxwell Fisheye lens, the variation range of the refractive index can be characterized as [1,$\sqrt 2$] and [1, 2], respectively. Hence, the farther away from the center of the lens, which is corresponding to the increasing of *θ*, the closer the values of the refractive index will be in orthogonal directions. In other words, when *θ* is large, the deviation value between *n*_*x*_ and *n*_*y*_ could be very small. Thus, *n*_*x*’*x*’_ and *n*_*y*’*y*’_ can still approximate to *n*_*x*_ and *n*_*y*_, respectively, and the cross couplings can also be diminished (i.e. *n*_*x*’*y*’_ ≈ *n*_*y*’*x*’_ ≈ 0). In brief, the values of *n*_*x*_ and *n*_*y*_ can be modulated independently for small incident angles; whereas the values of *n*_*x*_ and *n*_*y*_ should be designed approximately identical for the situations of large-angle incidence. From the analyses above, it is feasible to fulfill the requirements of a Luneburg-fisheye lens with an AM theoretically, applying the quasi-conformal mapping method^29,36^.

**Figure 3:**
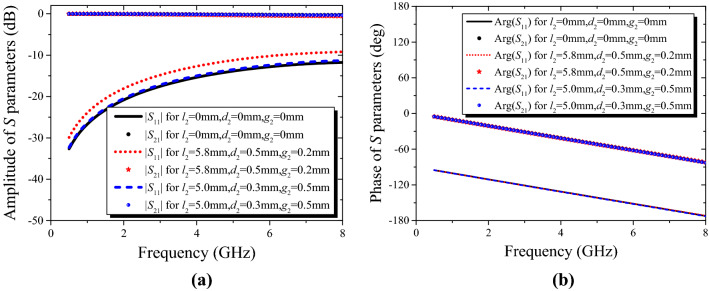
Variations of *S* parameters for the proposed anisotropic cells with different bottom dimensions (*l*_1_ = 4 mm, *d*_1_ = 0.35 mm, *g*_1_ = 0.4 mm). (**a**) Amplitude variation of *S* parameters. (**b**) Phase variation of *S* parameters.

To further validate the anisotropy of the proposed unit cell, the characteristics of the anisotropic cell are simulated by commercial software of CST Microwave Studio Suite. Provided that *x*-polarized incident waves propagate along *y*-axis as shown in Fig. 1, the variations of transmission and reflection coefficient versus frequency are plotted in Fig. 3. As can be seen from Fig. 3, despite changing the geometrical dimensions of the bottom structure arbitrarily, the differences between the values of *S*_11_ or *S*_21_ could be tiny, as long as the top structure remains unchanged (i.e. *l*_1_, *d*_1_, and *g*_1_ are fixed). Conversely, similar results will be achieved, given that the structure of the top and the bottom metallic layers is similar but oriented orthogonally. Obviously, the proposed unit cell can be anisotropic and the EM responses along *x*- and *y*-axis can be regulated independently as well. Moreover, magnitudes of the transmission coefficient *S*_21_ are all close to 0 in Fig. 3, indicating high transmission of the proposed AM (≥ 0.9).

**Figure 4:**
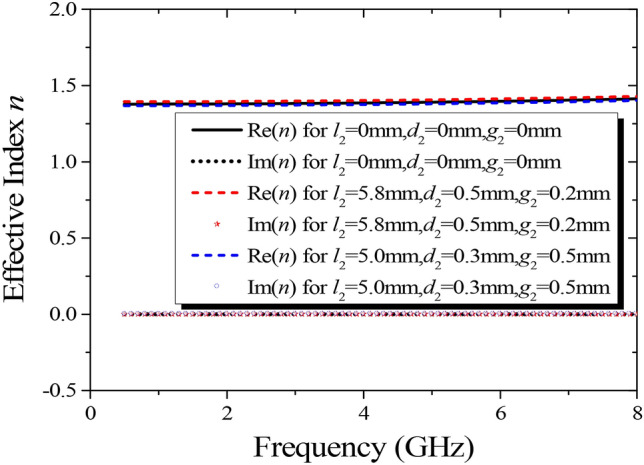
Variations of effective refractive index versus frequency when the dimensions of the top structure are fixed (*l*_1_ = 4 mm, *d*_1_ = 0.35 mm, *g*_1_ = 0.4 mm).

Applying effective medium theory, the effective refractive index of the proposed anisotropic cells can be retrieved by a *S*-parameter inversion method^37,38,39^. According to the correlative reflection and transmission coefficient shown in Fig. 3, the variations of effective refractive index for different bottom structural dimensions are displayed in Fig. 4. If the dimensions of top structure remain invariable, the effect on the retrieved parameters will be negligible, regardless of the sizes of the bottom structure, and the values of the effective refractive index are nearly constant in a wide frequency range as expected. In addition, the imaginary part of the extracted refractive index is roughly equal to 0, signifying low loss of the proposed lens.

**Figure 5:**
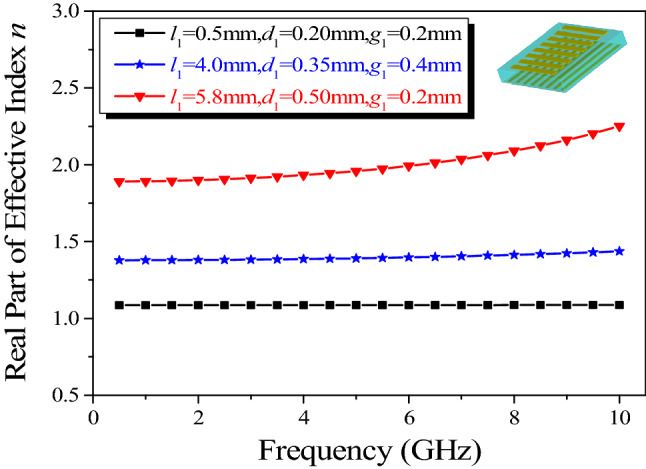
Variations of effective refractive index versus frequency when the dimensions of the bottom structure are fixed.

Another difficulty of realizing a broadband Luneburg-fisheye lens lies in the coverage of the effective refractive index. As can be seen from Fig. 3, the proposed anisotropic cell operates in a non-resonant region, leading to an inherently broadband property and low loss. Since the structure of the top layer is similar to that of the bottom layer, and the refractive index distributions in orthogonal directions can be adjusted independently, the refractive indexes of the anisotropic cells with varying structural parameters are extracted when the values of *l*_2_, *d*_2_, and *g*_2_ are fixed, as shown in Fig. 5. The variations of the effective refractive indexes versus frequency are minor, and by varying the geometries of the proposed anisotropic cell, the requirements of the refractive index distribution for the two lenses can be achieved in a wide band. The detailed variations of structural parameters are listed in Table 1. Accordingly, the corresponding values of the effective refractive index *n*_*x*_ for Luneburg lens can range from 1.04 to 1.40 in the band of 2 to 8 GHz, while, that of *n*_*y*_ for Maxwell fisheye lens can range from 1.04 to 1.99 in the band of 5 to 7 GHz.

**Table 1:** Geometry parameter variations of the proposed anisotropic cell (unit: mm).

Parameter	Value	Parameter	Value
*l*_1_	0.5–4.0	*l*_2_	0.5–5.8
*d*_1_	0.2–0.5	*d*_2_	0.2–0.5
*g*_1_	0.2–0.4	*g*_2_	0.2–0.5

### Design of the bifunctional Luneburg-fisheye lens

Sequel to the characteristics of the anisotropic cell, a broadband Luneburg-fisheye lens can be synthesized based on the specified refractive-index distributions of the two lenses.

**Figure 6:**
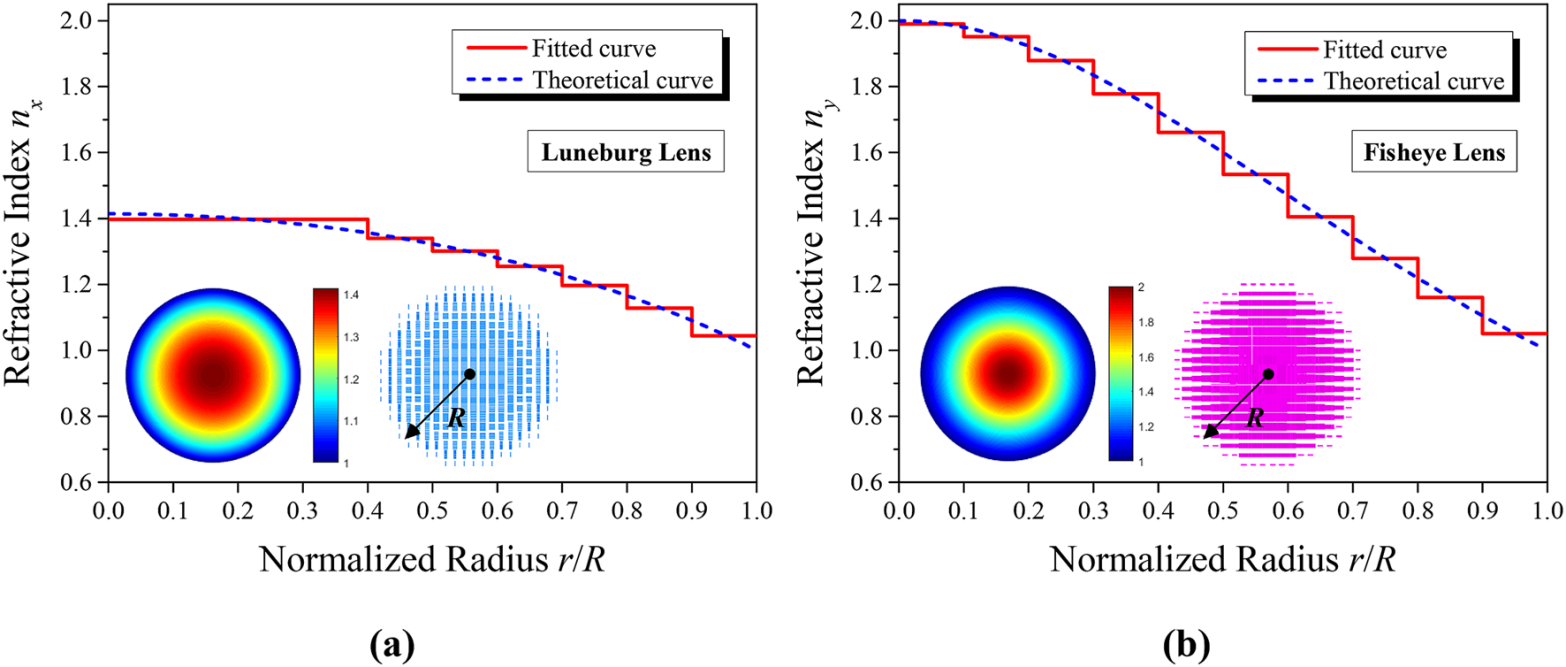
Comparison of the refractive index profiles between the theoretical and fitting distribution. In each graph, the left inset describes the ideal distribution of refractive index, and the right one exhibits the practical schematic of the synthesized lens. (**a**) Mapping of the Luneburg lens (top view). (**b**) Mapping of the Maxwell fisheye lens (bottom view).

The refractive index distribution of the Luneburg lens satisfies the following function of spatial position:

3 $$n_{x} (r) = \sqrt {2 - ({r \mathord{\left/ {\vphantom {r R}} \right. \kern-\nulldelimiterspace} R})^{2} } ,(0 \le r \le R)$$

where *R* is the radius of the lens, as shown in Fig. 6, and *r* is the radial distance away from the center of the lens. The refractive index distribution of the Maxwell fisheye lens obeys:

4 $$n_{y} (r) = {{n_{0} } \mathord{\left/ {\vphantom {{n_{0} } {[1 + ({r \mathord{\left/ {\vphantom {r R}} \right. \kern-\nulldelimiterspace} R})^{2} ]}}} \right. \kern-\nulldelimiterspace} {[1 + ({r \mathord{\left/ {\vphantom {r R}} \right. \kern-\nulldelimiterspace} R})^{2} ]}},(0 \le r \le R)$$

Here, *R* is the radius of the lens, and *r* is the radial coordinate as well. *n*_0_ represents the maximum refractive index at the center of the lens, whose value is set as 2 generally.

**Figure 7:**
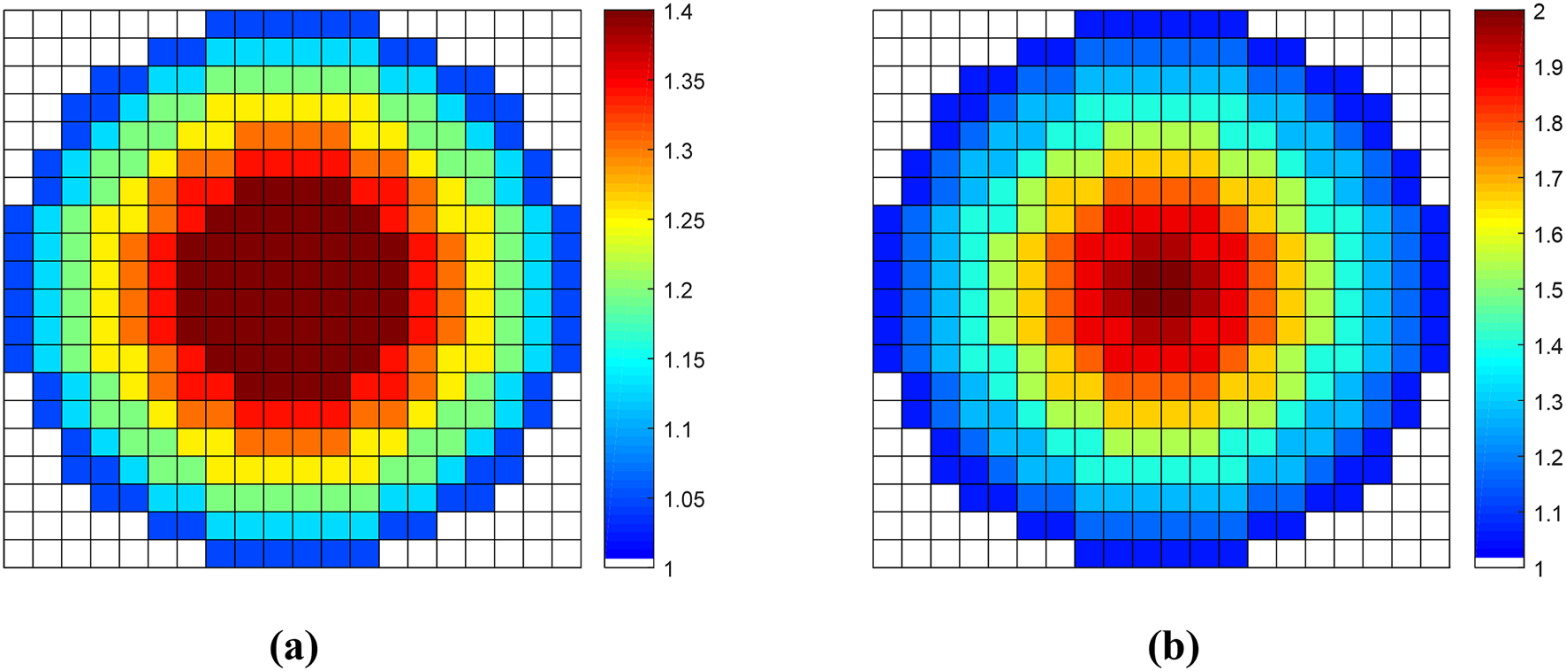
Discretized distribution of the refractive index. (**a**) Mapping of the Luneburg lens. (**b**) Mapping of the Maxwell fisheye lens.

As demonstrated in Fig. 6, stepped refractive index profiles are employed to approximate the theoretical ones calculated by (3) and (4). In order to integrate the functions of the two lenses into a single dielectric slab, an anisotropic metasurface consists of 20 × 20 cell blocks is designed, whose overall size is 120 mm × 120 mm × 1 mm (i.e. 3.2*λ* × 3.2*λ* × 0.027*λ* at 8 GHz). The discretized refractive-index distributions of the two lenses are displayed in Fig. 7, based on a method of quasi-conformal mapping. For practical implementation, the Luneburg lens and the fisheye lens is finally discretized into seven and ten concentric circular regions, respectively.

**Figure 8:**
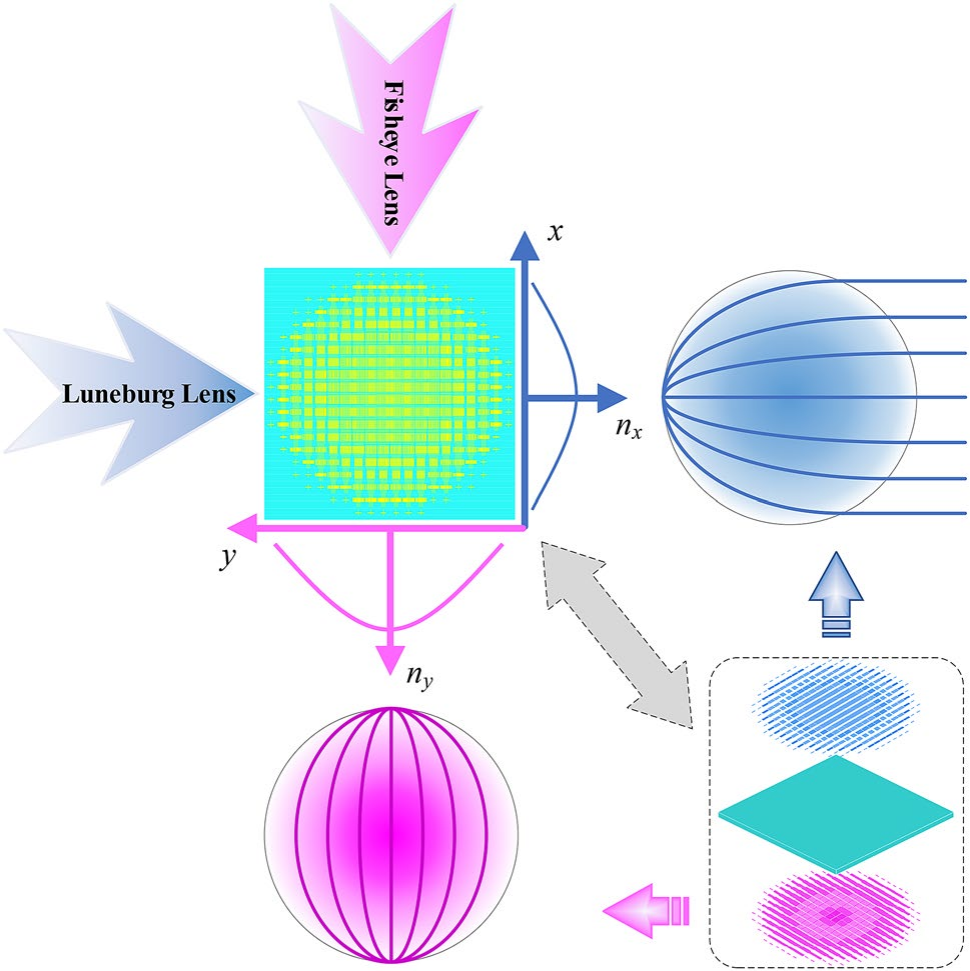
Illustration of the bifunctional Luneburg-fisheye lens synthesized by the proposed anisotropic cells.

Once the dimensions of the filling cells are ascertained in accordance with the refractive index distributions arranged in Fig. 7, a broadband bifunctional Luneburg-fisheye lens can be realized, as shown in Fig. 8. It can be seen clearly that the synthesized AM behaves as a Luneburg lens viewing from the horizontal orientation, while behaves as a Maxwell fisheye lens viewing from the vertical orientation. The metasurface on the top layer corresponds to the Luneburg lens (blue part), and the one on the bottom layer corresponds to the fisheye lens (pink part). Under the illumination of orthogonally polarized waves, the two lenses can achieve their distinct functions of beam focusing independently (see the blue and pink ray tracing, respectively).

### Simulated and experimental results of the bifunctional lens

**Figure 9:**
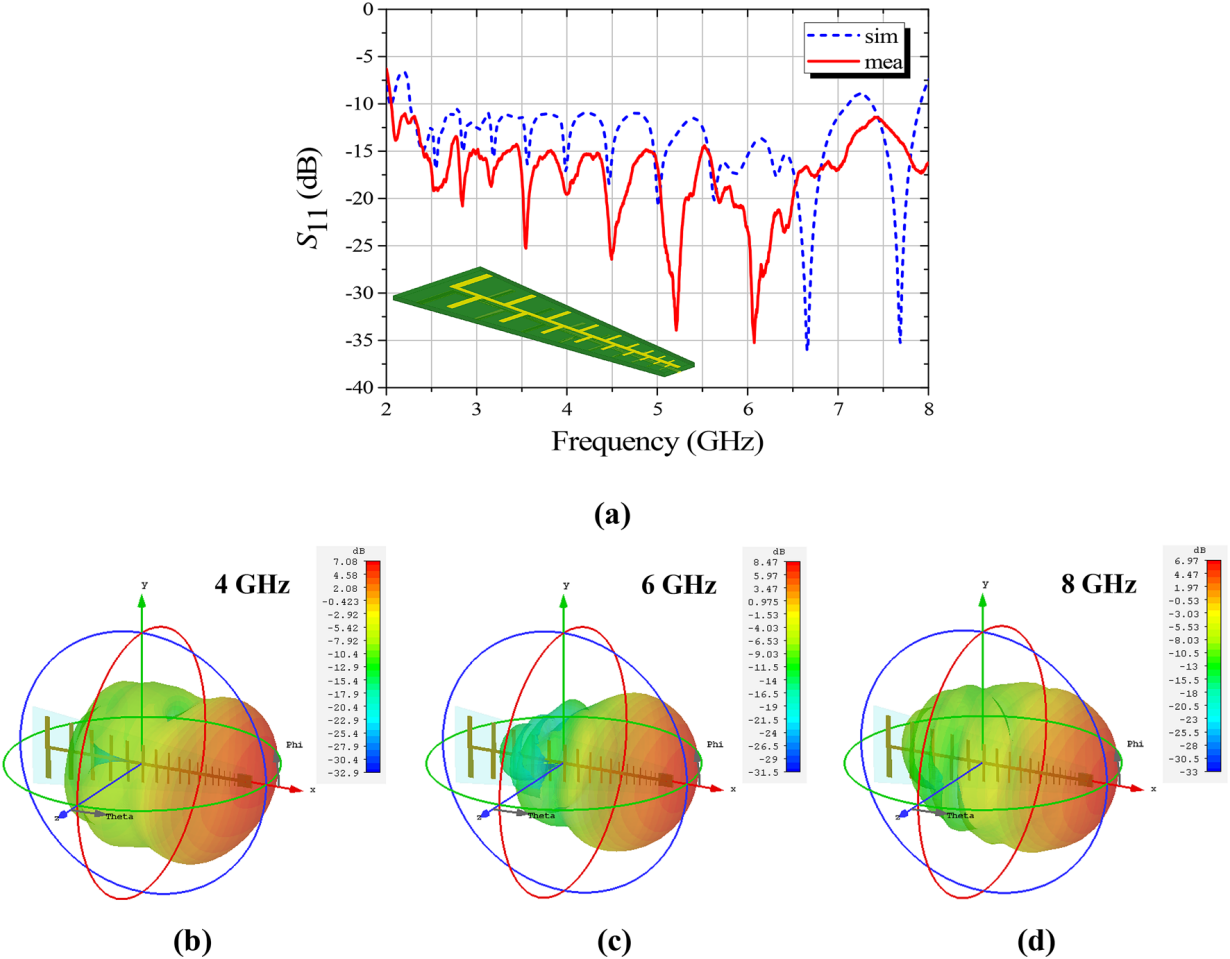
The performance of the feeding LPDA. (**a**) Simulated and measured *S*_11_ of the LPDA. The inset on the lower left is the model of the proposed LPDA. (**b**) 3-D radiation pattern of the LPDA at 4 GHz. (**c**) 3-D radiation pattern of the LPDA at 6 GHz. (**d**) 3-D radiation pattern of the LPDA at 8 GHz.

In consideration of the demand for broadband response and miniaturization, a planar log-periodic dipole antenna (LPDA) is designed as the feeding source, as shown in Fig. 9a. With which, the bifunctional Luneburg-fisheye lens can be excited by directional TE-mode surface waves, fulfilling the requirement of the proposed AM. The LPDA is printed on a commercial FR4 substrate (*ε*_*r*_ = 4.4, and tan *δ* = 0.02) with a thickness of 1 mm. The impedance characteristic (*S*_11_) and the radiation performance of the LPDA is detailedly presented in Fig. 9, respectively. As shown, the designed LPDA roughly operates over a wide frequency range from 2 to 8 GHz, and exhibits a desired performance of directional radiation along the central axis (see Fig. 9b–d).

**Figure 10:**
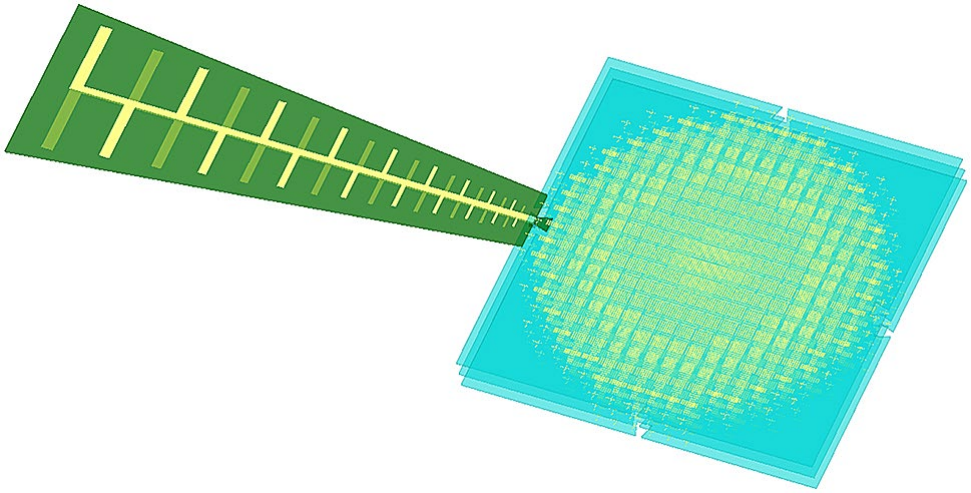
Schematic diagram of the whole system after assembly.

Aiming to improve the performance of the proposed bifunctional lens, a triple-layered structure is adopted, and the stacking AMs are spaced 6 mm apart from each other. The schematic diagram of the whole system after assembly is demonstrated in Fig. 10. Specifically, the feeding LPDA is coplanar with the middle AM, locating on the periphery of the metalens, and the central axis of the LPDA is aligned to that of the middle layer.

**Figure 11:**
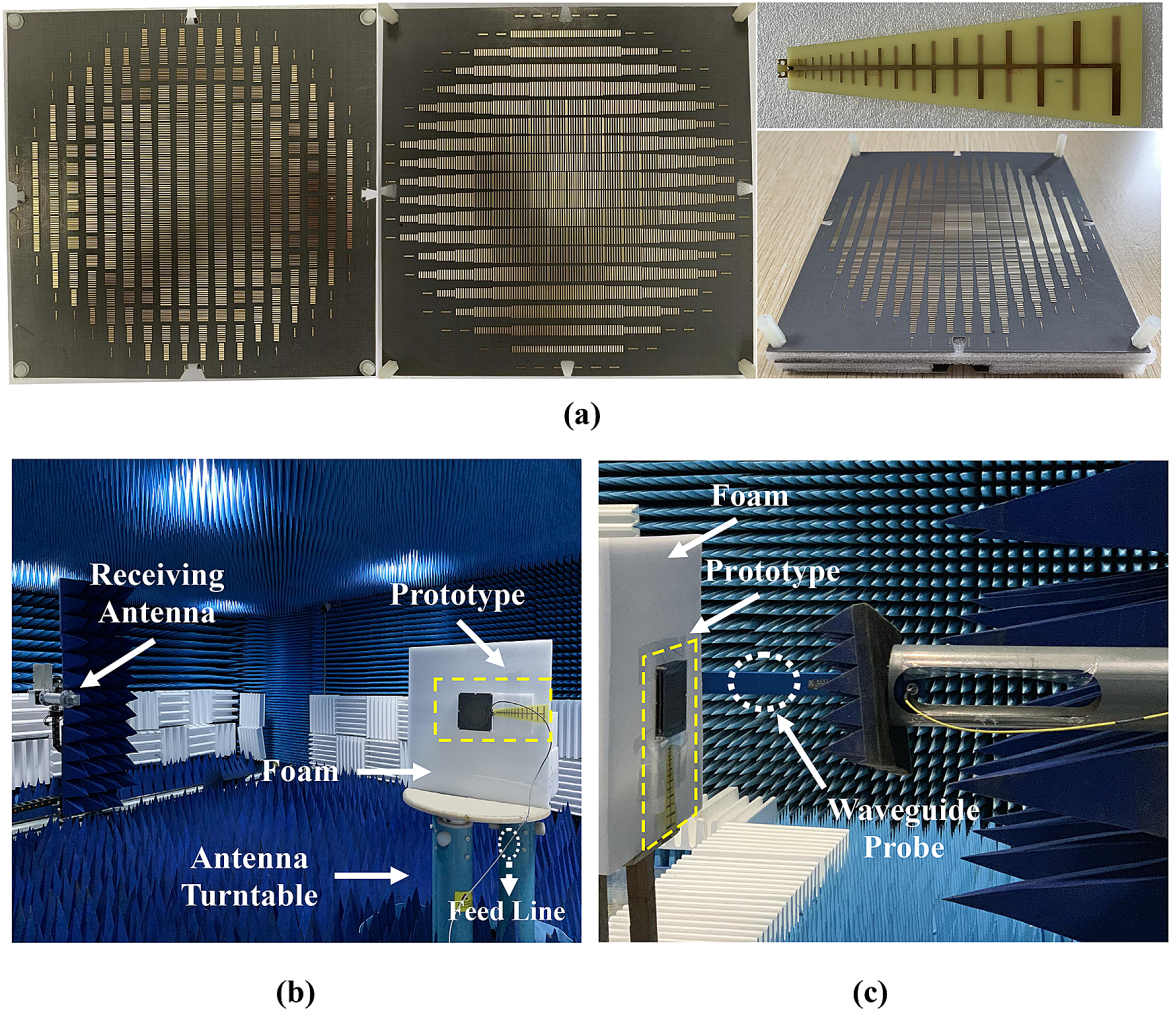
Photograph of the fabricated prototype and the experimental setup. (**a**) Prototypes of the Luneburg lens (top structure), the Maxwell fisheye lens (bottom structure), the feeding LPDA, and tri-layered metalens after assembly (from left to right). (**b**) Far-field setup. (**c**) Near-field setup.

Finally, to verify the performance of the proposed bifunctional metalens, a triple-layered prototype associated with the feeding LPDA is fabricated, assembled, and measured, as shown in Fig. 11. The three pieces of AM are sandwiched by foam spacers, whose relative permittivity and relative permeability are close to 1. The surroundings of far-field and near-field measurement are displayed in Fig. 11b, c, respectively. Besides, the scanning area of near field is 420 mm × 300 mm, which is sufficient to cover the entire lens system (marked by yellow rectangle), and the components of the tangential electrical fields on the metasurface are collected by a waveguide probe.

**Figure 12:**
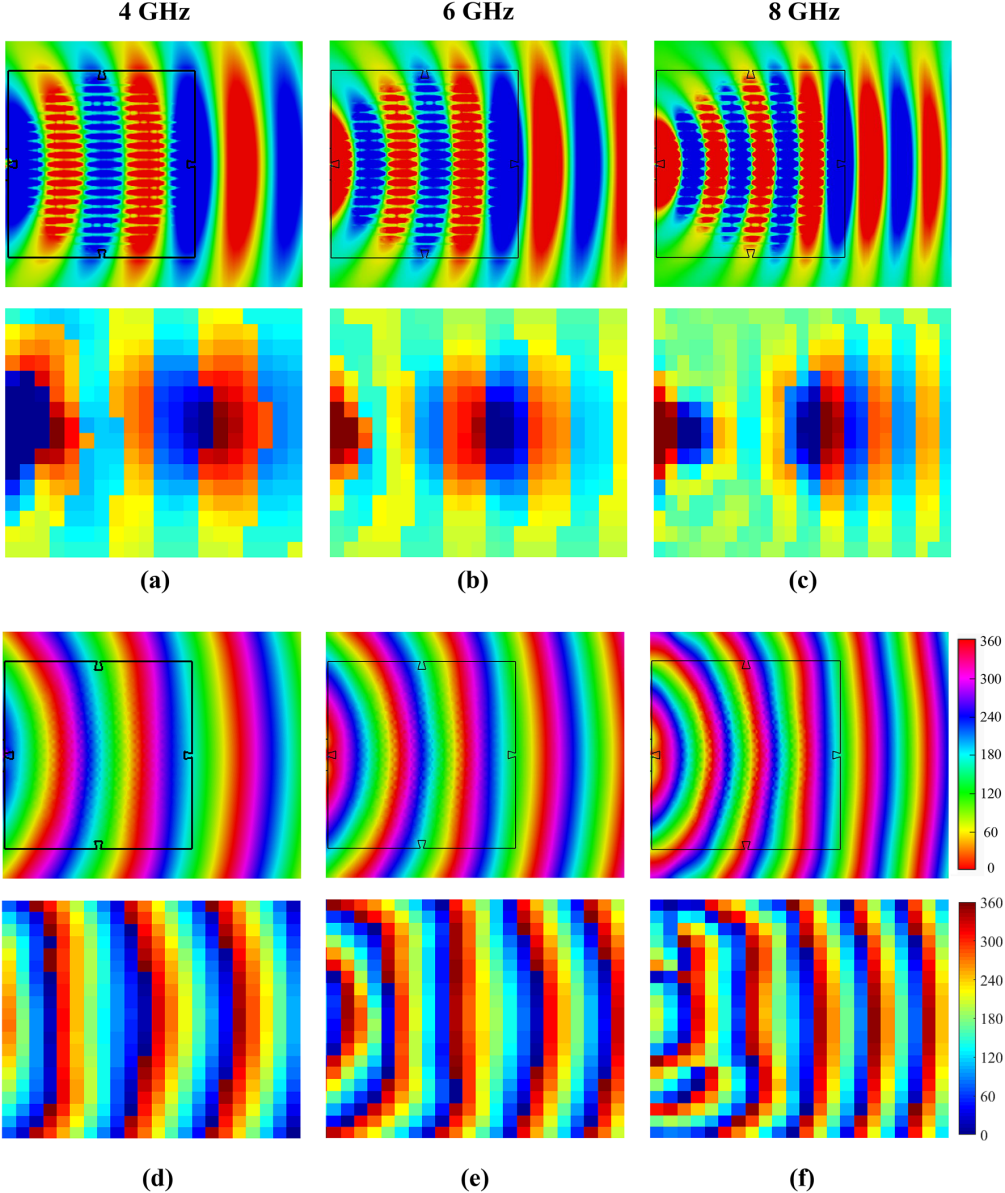
Comparisons of near electric-field distributions (*E*_*x*_) on *xoy*-plane between the simulated results (upper part of each subgraph) and measured results (bottom part of each subgraph) at 4, 6, and 8 GHz when the proposed bifunctional metalens serves as a Luneburg lens. (**a,b,c**) Amplitude distributions of simulated and measured electric field at 4, 6, and 8 GHz, respectively. (**d,e,f**) Phase distributions of simulated and measured electric field at 4, 6, and 8 GHz, respectively.

**Figure 13:**
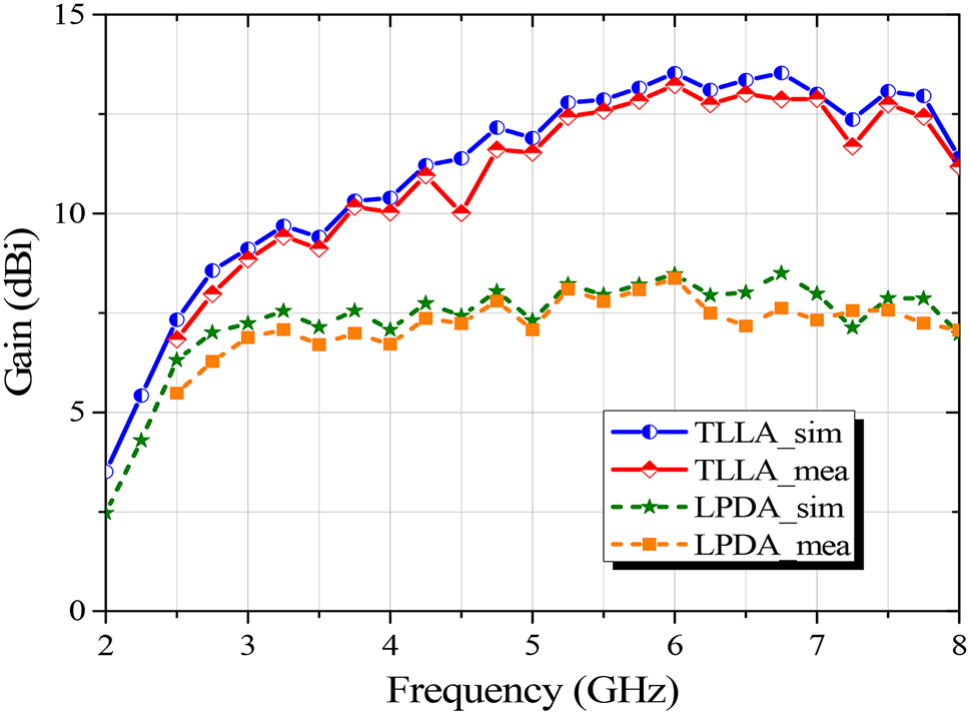
Comparison of the simulated and measured gains versus frequency when the proposed metalens serves as a Luneburg lens.

As described in Fig. 8, the proposed bifunctional metalens will function as a broadband Luneburg lens when the anisotropic metasurfaces are illuminated from *y* axis. The measured distributions of the instantaneous near electric field on the lens surface (*xoy*-plane) at 4, 6, and 8 GHz are compared with the numerical simulations successively, as demonstrated in Fig. 12. As can be seen from Fig. 12a–c, the measured electric-field distributions are in great agreement with the simulated ones, and the spherical waves radiated from the LPDA are dramatically transformed into quasi-plane waves, as predicted. To further reveal the performance of the metalens, the phase distributions of electric-field on *xoy*-plane are presented in Fig. 12d,e,f, as well. Likewise, it is obvious that the spherical wavefront can be converted to the quasi-planar wavefront in a broad band, and the effect of conversion is enhanced with the increase of frequency. For quantitative demonstration, simulated and measured gains versus frequency of the assembled tri-layered lens antenna (TLLA) and the feeding LPDA are plotted in Fig. 13. A significant gain enhancement can be observed from 2 to 8 GHz (relative bandwidth of 120%), which indicates a highly directional emission resulting from the Luneburg lens.

**Figure 14:**
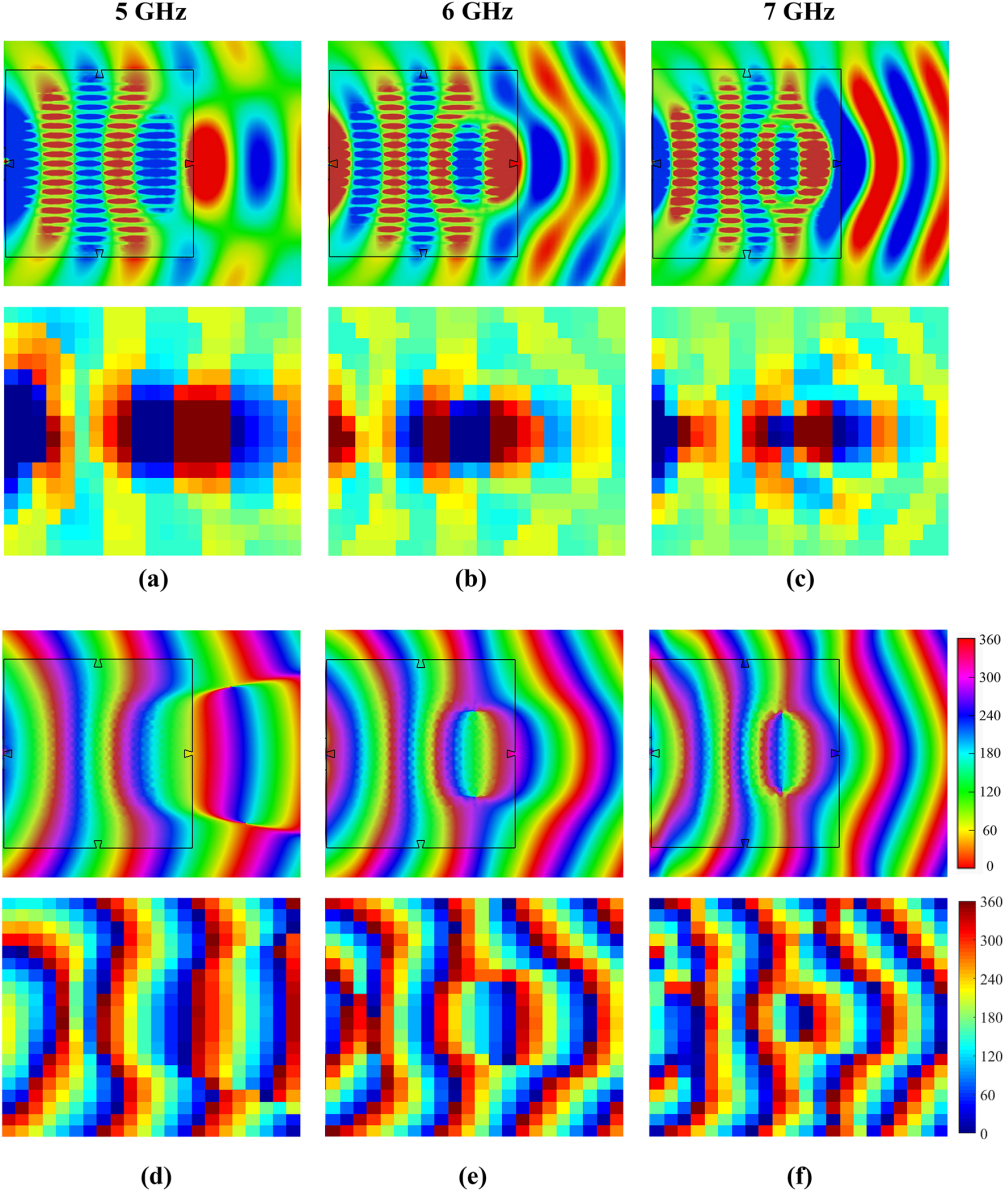
Comparisons of near electric-field distributions (*E*_*y*_) on *xoy*-plane between the simulated results (upper part of each subgraph) and measured results (bottom part of each subgraph) at 5, 6, and 7 GHz when the proposed bifunctional metalens serves as a fisheye lens. (**a,b,c**) Amplitude distributions of simulated and measured electric field at 5, 6, and 7 GHz, respectively. (**d,e,f**) Phase distributions of simulated and measured electric field at 5, 6, and 7 GHz, respectively.

Attributing to the anisotropy of the metasurface, the bifunctional metalens will function as a wide-band Maxwell fisheye lens when we rotate it 90 degrees clockwise with the location of the LPDA fixed. The comparisons of the instantaneous near electric fields on *xoy*-plane at 5, 6, and 7 GHz are demonstrated in Fig. 14. The experimental measurements agree well with the numerical simulations. Both amplitude and phase distributions of electric field show that the radiation emitted from the LPDA is focused on the opposite periphery of the metalens, and then propagates as spherical waves in a band of 5 to 7 GHz (relative bandwidth of 33.3%).

**Figure 15:**
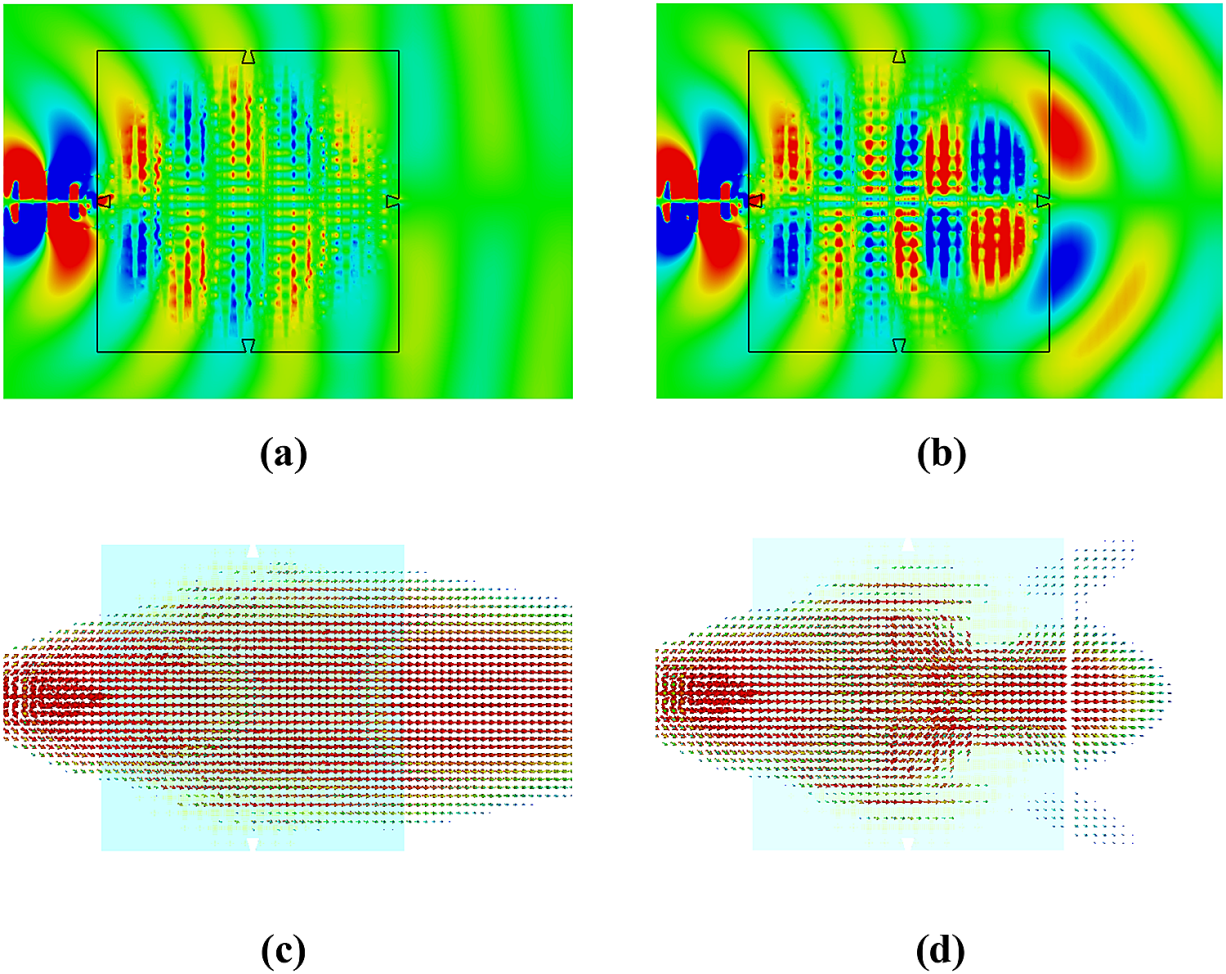
Simulated cross-polarized distributions of near electric-field regarding the bifunctional metalens on *xoy*-plane at 6 GHz. (**a**) The cross-polarized electric-field component (*E*_*y*_) of Luneburg lens. (**b**) The cross-polarized electric-field component (*E*_*x*_) of Maxwell fisheye lens. (**c**) Power-density distribution when illuminating from *y* axis (Luneburg lens). (**d**) Power-density of distribution when illuminating from *x* axis (fisheye lens).

In order to investigate the influence of cross-polarized electric fields on the performance of the bifunctional metalens, the simulated cross-polarized distributions of electric-field at 6 GHz for the Luneburg lens (*E*_*y*_) and Maxwell fisheye lens (*E*_*x*_) are illustrated in Fig. 15a,b, respectively. With respect to the TE-mode surface waves, the Poynting vector can be calculated as expression (5). According to the mechanism of the bifunctional metalens, the electric field *E*_*x*_ plays a dominant role in exciting the Luneburg lens, which is designed along *y*-axis. Similarly, the electric field *E*_*y*_ plays a dominant role in exciting the fisheye lens, which is designed along *x*-axis. Hence, it can be seen that the electric-field components of cross-polarization on the lens surface depicted in Fig. 15a,b are quite smaller than the corresponding components of co-polarization shown in Figs. 12 and 14. Moreover, compared with the theoretical predictions of beam path in Fig. 8, the power-density distributions in Fig. 15c,d show that the cross-polarized components of electric field do not much affect the power flows of the Luneburg and fisheye lenses as expected.

5 $$\mathop{S}\limits^{\rightharpoonup} = E_{y} H_{z} \cdot {{\mathop{e}^{\rightharpoonup}}_{x}}+ E_{x} H_{z} \cdot {{\mathop{e}^{\rightharpoonup}} _{y}}$$

## Conclusions

A broadband bifunctional Luneburg-fisheye lens based on anisotropic metasurface is proposed. The planar bifunctional metalens is synthesized by non-resonant anisotropic cells with low profile, so that it can achieve distinctly inhomogeneous distributions of refractive index in orthogonal directions, respectively. By virtue of the anisotropy, the bifunctional metalens can serve as a broadband Luneburg lens observing from the horizontal axis in the frequency range of 2 to 8 GHz, while as a wide-band Maxwell fisheye lens observing from the vertical axis in the operating band of 5 to 7 GHz. What’s more, our anisotropy-based approach is not only limited to an implementation of a Luneburg-fisheye lens, but can be flexibly applied to the realization of other broadband multifunctional metadevices operating in the region of microwave, optics, and even terahertz.
